# Complete Genome Sequence of *Mycoplasma pullorum* Isolated from Domestic Chickens

**DOI:** 10.1128/genomeA.01647-16

**Published:** 2017-02-23

**Authors:** Amanda Beylefeld, Celia Abolnik

**Affiliations:** Poultry Section, Department of Production Animal Studies, Faculty of Veterinary Science, University of Pretoria, Onderstepoort, South Africa

## Abstract

The 1,007,172-bp complete genome of *Mycoplasma pullorum* strain B359_6, isolated from domestic chickens, has been sequenced, assembled, and annotated.

## GENOME ANNOUNCEMENT

Mycoplasmas evolved through reductive evolution from Gram-positive bacteria, resulting in the smallest known self-replicating prokaryotes. These prokaryotes are dependent on their hosts for most of their nutrients due to the loss of most of their protein-coding genes (1). *Mycoplasma pullorum* was first characterized in 1982 as an aerobic and anaerobic fermenter of glucose, but it is unable to reduce tetrazolium chloride or hydrolyze arginine or urea, and lacks phosphatase activity (2). *M. pullorum* is predominately isolated from chickens, but has also been found in turkeys, pheasants, and partridges (3–5).

*M. pullorum* strain B359_6 was isolated in February 2015 from a 72-week-old layer hen showing clinical signs of mycoplasma infection, which included breathing difficulty, rales, and nasal discharge. The organism was cultured in mycoplasma broth, and the DNA was extracted from 200 mL of culture, as previously described (6). Genomic DNA was sequenced on both the Ion Torrent (University of Pretoria) and Illumina MiSeq (Inqaba Biotech [Pty] Ltd., Pretoria) platforms. The Ion Torrent data set comprised 4,048,280 reads with an average length of 176.74 nucleotides (nt) and approximately 700× coverage, and the Illumina MiSeq data set contained 332,818 reads with an average length of 185.12 nt and approximately 60× coverage. The genome was assembled *de novo* in CLC Genomics Workbench version 8.5.1 to create two contig sets. The Ion Torrent data set was also submitted to IonGap (http://iongap.hpc.iter.es) for *de novo* assembly to create a third contig set (7). The three contig data sets were then aligned in the CLC Genome Finishing Tool version 1.5.4 and systematically joined by visual inspection. The 16S rRNA gene of strain B359_6, identified using RNAmmer (http://www.cbs.dtu.dk/services/RNAmmer), as well as the 16S rRNA genes from reference sequences retrieved from GenBank, were used to reconstruct the 16S rRNA phylogeny (8). Parsimony, maximum likelihood, and Bayesian inference phylogenetic trees were constructed in PAUP version 4, PhyML version 3.0, and MrBayes version 3.2.6, respectively, and strain B359_6 was identified as *M. pullorum* (9–12). Strain B359_6 had 99.80% nucleotide sequence identity to the 16S rRNA gene of *M. pullorum* strain ATCC 33553.

Annotation of the 1,007,172-bp circular genome of the *M. pullorum* strain B359_6 genome was completed in the NCBI Prokaryotic Genome Annotation Pipeline (PGAP) (13). The genome has a G+C content of 29.07% and molecular weight of 6 × 10^8^ Da, and contains 763 coding genes, two sets of the 16S and 23S rRNA genes, and 34 tRNAs. This sequence represents the first complete, annotated genome for *M. pullorum*.

### Accession number(s).

The whole-genome sequence for *M. pullorum* strain B359_6 was submitted to GenBank under accession number CP017813.

## References

[1] Citti C, Blanchard A 2013 Mycoplasmas and their host: emerging and re-emerging minimal pathogens. Trends Microbiol 21:196–203. doi:10.1016/j.tim.2013.01.003. 23419218

[2] Jordan FTW, Ernø H, Cottew GS, Hinz KH, Stipkovits L 1982 Characterization and taxonomic description of five mycoplasma serovars (serotypes) of avian origin and their elevation to species rank and further evaluation of the taxonomic status of *Mycoplasma synoviae*. Int J Syst Bacteriol 32:108–115. doi:10.1099/00207713-32-1-108.

[3] Benčina D, Dorrer D, Tadina T 1987 *Mycoplasma* species isolated from six avian species. Avian Pathol 16:653–664. doi:10.1080/03079458708436413. 18766652

[4] Moalic PY, Kempf I, Gesbert F, Laigret F 1997 Identification of two pathogenic avian mycoplasmas as strains of *Mycoplasma pullorum*. Int J Syst Bacteriol 47:171–174. doi:10.1099/00207713-47-1-171. 8995821

[5] Bradbury JM, Yavari CA, Dare CM 2001 Mycoplasmas and respiratory disease in pheasants and partridges. Avian Pathol 30:391–396. doi:10.1080/03079450120066395. 19184924

[6] Abolnik C, Gouws J 2014 Extended survival times of *Mycoplasma gallisepticum* and *Mycoplasma synoviae* on kanekalon synthetic hair fibres. Poult Sci 93:8–11. doi:10.3382/ps.2013-03457. 24570416

[7] Baez-Ortega A, Lorenzo-Diaz F, Hernandez M, Gonzalez-Vila CI, Roda-Garcia JL, Colebrook M, Flores C 2015 IonGAP: integrative bacterial genome analysis for Ion Torrent sequence data. Bioinformatics 31:2870–2873. doi:10.1093/bioinformatics/btv283. 25953799

[8] Lagesen K, Hallin P, Rødland E, Stærfeldt H-H, Rognes T, Ussery D 2007 RNAmmer: consistent annotation of rRNA genes in genomic sequences. Nucleic Acids Res 35:3100–3108. doi:10.1093/nar/gkm160. 17452365 PMC1888812

[9] Swofford DL 2003 PAUP*: phylogenetic analysis using parsimony (and other methods), version 4. Sinauer Associates, Sunderland, MA.

[10] Guindon S, Dufayard JF, Lefort V, Anisimova M, Hordijk W, Gascuel O 2010 New algorithms and methods to estimate maximum-likelihood phylogenies: assessing the performance of PhyML 3.0. Syst Biol 59:307–321. doi:10.1093/sysbio/syq010. 20525638

[11] Huelsenbeck JP, Ronquist F 2001 MrBayes: Bayesian inference of phylogenetic trees. Bioinformatics 17:754–755. doi:10.1093/bioinformatics/17.8.754. 11524383

[12] Ronquist F, Huelsenbeck JP 2003 MrBayes 3: Bayesian phylogenetic inference under mixed models. Bioinformatics 19:1572–1574. doi:10.1093/bioinformatics/btg180. 12912839

[13] Tatusova T, DiCuccio M, Badretdin A, Chetvernin V, Nawrocki EP, Zaslavsky L, Lomsadze A, Pruitt KD, Borodovsky M, Ostell J 2016 NCBI prokaryotic genome annotation pipeline. Nucleic Acids Research 44:6614–6624. doi:10.1093/nar/gkw569. 27342282 PMC5001611

